# Absence of gemin5 from SMN complexes in nuclear Cajal bodies

**DOI:** 10.1186/1471-2121-8-28

**Published:** 2007-07-18

**Authors:** Le thi Hao, Heidi R Fuller, Le Thanh Lam, Thanh T Le, Arthur HM Burghes, Glenn E Morris

**Affiliations:** 1 Wolfson Centre for Inherited Neuromuscular Disease, LMARC Building, RJAH Orthopaedic Hospital, Oswestry, SY10 7AG, UK. and Institute of Science and Technology in Medicine, Keele University, ST4 7QB, UK; 2 Dept. of Medical Biochemistry, Ohio State University, Columbus, OH 43210, USA; 3 Center for Molecular Neurobiology, Ohio State University, Columbus, OH 43210, USA

## Abstract

**Background:**

Spinal muscular atrophy is caused by reduced levels of the survival of motor neurons (SMN) protein. SMN is found in large complexes with Sm proteins and at least eight other proteins, including seven "gemins". These complexes are involved in the assembly of snRNPs in the cytoplasm and their transport into the nucleus, but the precise roles of the individual protein components are largely unknown.

**Results:**

We have investigated the subcellular distribution of gemins using novel antibodies against gemins 3–7, and existing mAbs against SMN, gemin2, unrip, fibrillarin and profilin II. Most gemins were equally distributed between nuclear and cytoplasmic fractions of HeLa cells, but gemin5 and unrip were more abundant in the cytoplasm. In a cytoplasmic extract obtained by mild disruption of HeLa cells, nearly all the SMN and gemins 2–4 were in large complexes, but most of the gemin5 sedimented separately with a lower S value. Most of the unrip sedimented with gemins 6 and 7 near the top of the sucrose density gradients, separate from both SMN and gemin5. Anti-SMN mAbs pulled down gemin5 from cytoplasmic extracts, but not from nuclear extracts, and gemin5 did not co-sediment with large SMN complexes in nuclear extracts. These data suggest that gemin5 is easily detached from SMN-gemin complexes in the nucleus. By immuno-histochemistry, gemin5 was rarely detectable in nuclear gems/Cajal bodies, although it was accessible to antibody and easily detectable when present. This suggests that gemin5 is normally absent from SMN complexes in these nuclear storage sites.

**Conclusion:**

We conclude that SMN complexes usually exist without gemin5 in nuclear gems/Cajal bodies. Gemin5 is believed to be involved in capturing snRNA into SMN complexes in the cytoplasm for transport into the nucleus. We hypothesize that gemin5, though present in the nucleus, is no longer needed for SMN complex function during the time these complexes are stored in gems/Cajal bodies.

## Background

The SMN protein forms a stable complex with a group of proteins named gemins [reviewed in [1,2]]. The gemins colocalize with SMN in gems/Cajal bodies (CBs) and are also present throughout the cytoplasm and in the nucleoplasm [1], although gemin4 has also been reported in the nucleolus [3]. An early view of the structure of the SMN complex was that gemins 2, 3, 5 and 7 bind directly to SMN, while gemins 4 and 6 associated by interaction with gemins 3 and 7, respectively [4]. It was later shown that a novel protein, gemin8, mediates the binding to SMN of the subcomplex of gemins 6 and 7 and a protein called unrip [5]. A recent study suggests that SMN interacts directly only with gemins 2, 3 and 8, while the subcomplex of gemin7 with gemin6 binds through gemin8, unrip binds through gemin7, gemin5 binds through gemin2, and gemin4 binds to both gemins 3 and 8 [2]. SMN complexes clearly have an important and well-documented role in both assembly of cytoplasmic snRNPs and their transport to the nucleus [5-8]. However, a significant amount of SMN is also found in the cytoplasm of motor neuron axons, suggesting that SMN may have motor neuron-specific functions independent of snRNP assembly [9-17].

Immunopurification of a 300-kDa SMN-gemin2 complex showed that it also contained spliceosomal snRNP core proteins B/B', D1, D2, D3, E, F and G [6]. There is some controversy in the literature on whether there is an SMN interaction site for Sm core proteins near the C-terminus (residues 240–267; [6]) or at residues 120–160 in the exon3-encoded tudor domain [18]. Charroux et al [3] described an 800 kDa complex that included SMN, gemin2 and gemins 3 and 4. Gemins 3 and 4 were also found without SMN in a separate 15S complex that contains eukaryotic initiation factor 2C and numerous microRNAs [19]. Meister et al [20] isolated two distinct SMN complexes from HeLa nuclei, NSC1 and NSC2, that migrated in sucrose gradients at 20S and 18S respectively. NSC1 was U snRNA-free, but contained at least 10 proteins, including SMN, gemin2, gemins 3 and 4 and Sm proteins D1, D2 and F. They later described a complex in both nucleus and cytoplasm that contains all gemins and Sm core proteins, plus unrip and hsc70 [21]. Unrip is an interacting partner of unr, a cytoplasmic RNA binding protein involved in the translation of viral RNAs [22].

The functions of individual proteins in SMN complexes and how they contribute to the overall function of the complex remains unclear. A role for gemin2 in the oligomerization of the SMN complex was recently shown [24]. Gemin3 is a 103 kDa RNA helicase that binds to Epstein-Barr virus-encoded nuclear antigens [25]. Its binding partner, gemin4, binds to protein phosphatase 4, overexpression of which can affect the localization of newly formed snRNPs in HeLa cells [26] and SMN phosphorylation is important for U snRNP assembly [27]. Gemin4 was also found to interact with galectins 1 and 3, which are involved in mRNA splicing [28]. Gemin5 has thirteen WD domains which, in other proteins, form "propeller" structures involved in protein-protein interactions [29]. Gemins 6 and 7 fold together to form a structure that resembles Sm core protein dimers [30]. It has been suggested that gemin6 and gemin7 play a role in organizing Sm proteins for assembly onto snRNAs by serving as an Sm-like dimer surrogate around which individual Sm proteins are arranged for binding to the Sm site [30]. The gemin 6–7–8-unrip subcomplex is required for recruiting Sm core proteins to SMN complexes [5].

We have produced novel panels of antibodies against gemins and used them to investigate the subcellular and molecular distribution of gemins in the cell. The studies have revealed a striking deficiency of gemin5 in nuclear gems/CBs and have shown that a large proportion of gemin5 exists separately from SMN complexes.

## Results

### Characterization of antibodies

**Figure 1:**
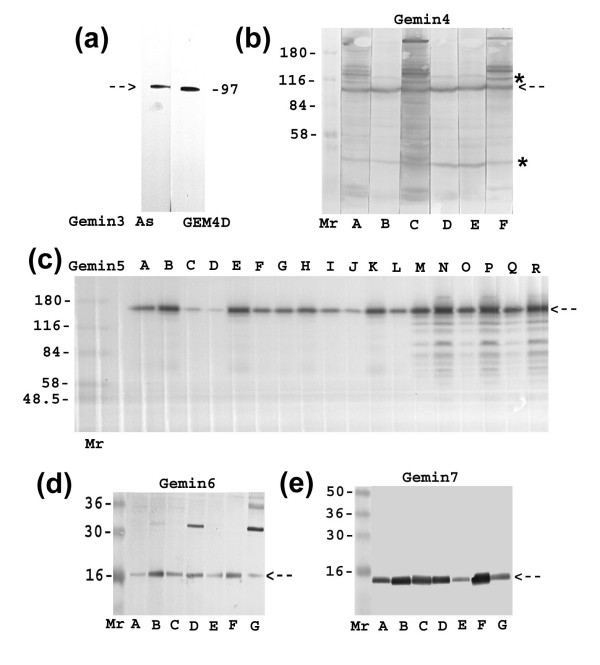
**Characterization of gemin antibodies by western blots of total HeLa cell proteins**. An SDS extract of HeLa cells was run as a horizontal strip alongside Mr markers on SDS-PAGE and transferred to nitrocellulose membranes by electroblotting. 7% acrylamide was used for (a)-(c) with prestained Sigma markers and 14% for (d) and (e) with prestained SeeBlue markers. The blot was either cut into vertical lanes or, in (c) only, used directly on a 28-lane miniblotter apparatus. In each case, the main antigen band is shown as a broken arrow. (a) gemin3 antiserum stains a single protein of about 102 kD (97 kD gemin4 is shown as a marker). (b) the panel of 6 gemin4 mAbs all stain a band of 97 kD, but 3 of them (GEM4A, C and F) cross-react with several higher Mr proteins. Non-specific bands (*) are due to the secondary antibody system. (c) the 12 mAbs against the gemin5 N-terminal region stain a single band consistent with the 167 kD expected for gemin5 and the 6 mAbs against the C-terminal region stain the same band, together with a ladder of smaller bands that may be degradation products. (d) The 7 gemin6 mAbs all stain a band of 16 kD, but 2 of them cross-react with a 32 kD band. (e) All 7 gemin7 mAbs stain a single band of 15 kD.

For this study, we produced new panels of monoclonal antibodies (mAbs) against gemins 4, 5, 6 and 7 (Table 1). We also produced a new polyclonal antibody against gemin3 to complement mAbs against SMN and gemin2 described previously [31]. Our first step was to demonstrate the specificity of the new antibodies by western blots of total HeLa cell proteins (Fig. 1). The anti-gemin3 serum gave a single band close to the expected size of 102 kD; the GEM4D mAb against gemin4 (97 kD) is shown alongside for comparison. Three gemin4 mAbs (GEM4B, D and E) recognise a single band, apart from non-specific bands caused by the secondary antibodies, but the other three mAbs stain extra bands of higher Mr. The more specific mAbs were used in subsequent studies. All the gemin5 mAbs stained a band consistent with the predicted Mr of 167 kD. Only the C-terminal gemin5 mAbs (GEM5M-R) also stained a ladder of lower Mr bands down to about 60 kD; this may be due to partial degradation of gemin5 in its N-terminal "WD-propeller" region. The N-terminal mAbs (GEM5A-L) were not useful for immunolocalization because they appear to recognize only denatured and unfolded protein on western blots (Table 1). In support of this, we were able to map the epitope for GEM5J to amino-acids 66–71 (RVSGFT) using phage-displayed peptide libraries as described previously [32] and this sequence is part of the highly-structured "WD-propeller" in the native state. Gemin6 mAbs stained a protein of 16 kD and gemin7 mAbs a protein of 15 kD, both migrating slightly faster than predicted by their amino-acid sequence. Two of the gemin6 mAbs cross-reacted with higher Mr proteins, notably one of about 32 kD. The more specific GEM6F was used in subsequent studies. We also used antibodies against other proteins known to interact with SMN including fibrillarin [33], unrip and profilin II [17].

**Table 1:** Characterization of gemin monoclonal antibodies.

**Clone**	**Name mAbs**	**Sub class**	**Western Blot**					**IMF**	
			
			**Recom**	**HeLa**	**COS7**	**Pig**	**Fish**	**HeLa**	**COS7**
1A8	GEM4A	IgA	**+**	**++**	**++**	**+**	-	**n.d.**	**n.d.**
1G4	GEM4B	IgG1	**+**	**++**	**++**	**+**	-	**+**	**+**
9E6	GEM4C	IgG2b	**+**	**++**	**++**	**+**	-	**+**	**+**
2H8	GEM4D	IgG1	**+**	**++**	**++**	**+**	-	**+**	**+**
7B5	GEM4E	IgG2a	**+**	**++**	**++**	**+**	-	**+**	**+**
2A2	GEM4F	IgG2b	**+**	**++**	**++**	**+**	-	**+**	**+**
1A5	GEM5A	IgG1	**+**	**++**	**+**	**-**	-	**-**	**-**
1B1	GEM5B	IgG2a	**+**	**++++**	**+++**	**-**	+	**-**	**-**
1C10	GEM5C	IgG2b	**+**	**+**	**+**	**+**	-	**-**	**-**
2A12	GEM5D	IgG2b	**+**	**+**	**+**	**+**	-	**-**	**-**
2B11	GEM5E	IgG2a	**+**	**++++**	**++**	**-**	+	**-**	**-**
5C3	GEM5F	IgG2a	**+**	**++**	**++**	**-**	-	**-**	**-**
6C4	GEM5G	IgG1	**+**	**++**	**++**	**-**	+	**-**	**-**
6E2	GEM5H	IgG2b	**+**	**+++**	**+++**	**-**	+	**-**	**-**
7A1	GEM5I	IgG1	**+**	**++**	**++**	**-**	+	**-**	**-**
7F1	GEM5J	IgG1	**+**	**+**	**+**	**-**	-	**-**	**-**
8C7	GEM5K	IgG2b	**+**	**++++**	**+++**	**-**	+	**-**	**-**
8E7	GEM5L	IgG2b	**+**	**++**	**+**	**-**	+	**-**	**-**
1E12	GEM5M	IgG1	**+**	**+++**	**++**	**++**	-	**+**	**+**
2E6	GEM5N	IgG1	**+**	**++++**	**+++**	**++**	-	**+**	**+**
3E11	GEM5O	IgG1	**+**	**+++**	**++**	**++**	-	**+**	**+**
3G2	GEM5P	IgG1	**+**	**++++**	**+++**	**++**	-	**+**	**+**
4G7	GEM5Q	IgG1	**+**	**+++**	**+++**	**++**	-	**+**	**+**
6G5	GEM5R	IgG1	**+**	**++++**	**+++**	**++**	-	**-**	**-**
2C11	GEM6A	IgG2a	**+**	**+**	**+**	**-**	-	**+**	**+**
4H6	GEM6B	IgG2a	**+**	**++**	**+**	**+**	-	**+**	**+**
5E9	GEM6C	IgG1	**+**	**+**	**+**	**-**	-	**-**	**-**
6F5	GEM6D	IgG2a	**+**	**+**	**+**	**+**	-	**+**	**+**
8A9	GEM6E	IgG2a	**+**	**+**	**+**	**-**	-	**+**	**+**
8D8	GEM6F	IgG2a	**+**	**++**	**+**	**+**	-	**+**	**-**
3F12	GEM6G	IgG2b	**+**	**+**	**+**	**-**	-	**+**	**-**
2A11	GEM7A	IgG1	**+**	**+**	**+**	**-**	-	**+**	**+**
6A2	GEM7B	IgG1	**+**	**+++**	**++**	**-**	-	**+**	**+**
6A9	GEM7C	IgG1	**+**	**++**	**+**	**-**	-	**-**	**-**
7A12	GEM7D	IgG1	**+**	**++**	**+**	**-**	-	**+**	**+**
8D8	GEM7E	IgG1	**+**	**+**	**+**	**-**	-	**+**	**+**
8H1	GEM7F	IgG1	**+**	**+++**	**++**	**+**	-	**+**	**+**
10C12	GEM7G	IgG1	**+**	**+**	**+**	**-**	-	**+**	**+**

### By immunolocalization, gemin5 is absent from nuclear gems/Cajal bodies in most cells

**Figure 2:**
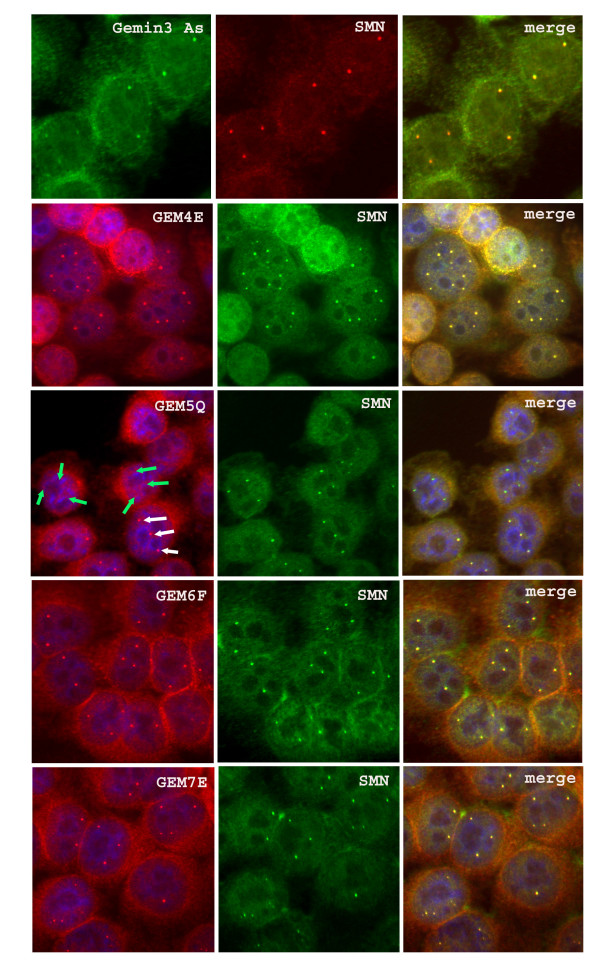
**Gemins 3, 4, 6 and 7 colocalise with SMN in nuclear gems/CBs, but gems/CBs in most cells lack gemin5**. HeLa cells grown on coverslips were fixed with 1% formalin in PBS and permeabilized with 1% Triton X-100. Gemins 4, 5, 6 and 7 were identified using GEM4E, GEM5Q, GEM6F and GEM7E, respectively in a double label with SMN rabbit antiserum. Typical fields are shown, except that an unusual field containing several cells with gemin5-positive gems/CBs was chosen for GEM5Q. The anti-gemin3 rabbit antiserum was double-labelled with MANSMA1 mAb against SMN. Alexa fluor 546 goat anti-mouse IgG (red) and Alexa fluor 488 goat anti-rabbit IgG (green) were used as second antibodies. DAPI (blue) was used to counterstain the nuclei. (See text for white and green arrows).

**Figure 3:**
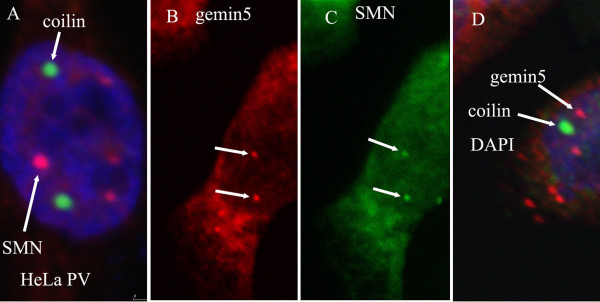
**In Hela PV cells, which have separate gems and CBs, gemin5 colocalizes with gems, not CBs, in those rare cells that do have gemin5-positive nuclear bodies**. HeLa strain PV cells grown on coverslips were fixed with 1% formalin in PBS and permeabilized with 1% Triton X-100. (A) Separation of gems (red: MANSMA1 mAb and Alexa-546 anti-mouse Ig) and CBs (green: rabbit anti-coilin and FITC anti-rabbit Ig) in HeLa PV, counterstained with DAPI (blue). Rare gemin5-positive nuclear bodies were double-labelled with (B) mAb GEM5P and Alexa-546 anti-mouse Ig and (C) rabbit anti-SMN and FITC anti-rabbit Ig to show colocalization of SMN and gemin5 (white arrows). (D) is an overlay from a double label with GEM5P (red) and rabbit anti-coilin (green) with a DAPI counterstain (blue), showing no colocalization.

We next needed to confirm that all the gemin antibodies stained gems or Cajal bodies (gems/CBs), as previously reported, and we found that most gemins colocalize with SMN in nuclear gems/CBs in HeLa cells, the exception being gemin5 (Fig. 2). Although colocalization of SMN and gemin5 was observed in some cells (white arrows), gemin5 was absent from gems/CBs in other cells in the same field of view (green arrows). Gems/CBs also lacked gemin5 in human Ntera-2 cells and skin fibroblasts. This was the first indication that gemin5 may not always be associated with SMN. Although gemin5 mAb does not stain any SMN-positive gems/CBs in most nuclei, it stains the normal number of gems in the rare gemin5-positive nuclei. This "all or nothing" staining of gemin5 is illustrated in Table 2 where only positive nuclei were counted and the average gem count was 2.41 for gemin5 compared with 2.5–2.6 for SMN and other gemins. To determine whether gemin5 is associating with gems or CBs in the rare gemin5-positive nuclei, we used the HeLa PV cell line in which gems and CBs are separate (Fig. 3A) and found that gemin5 colocalizes with SMN in gems (Fig. 3B &3C), but not with coilin in CBs (Fig. 3D).

**Table 2:** Gemin5 staining is "all or nothing".

mAb	Number of nuclei with gems/CBs	Total number of gems/CBs	Average number of gems/CBs per positive nucleus
MANSMA1	113	290	2.57
MANSIP1A	93	242	2.60
GEM4E	103	257	2.50
GEM5Q	27	65	2.41
GEM6F	114	286	2.51
GEM7E	111	280	2.52

In those rare HeLa cell nuclei that have gemin5-positive gems/CBs, nearly all gems/CBs were positive for gemin5. HeLa cell cultures on glass coverslips were fixed with 50:50 acetone: methanol, dried and labelled individually with gemin mAbs. Only nuclei with positive gem/CB staining were counted.

### Unlike other gemins, gemin5 is not co-regulated with SMN

**Figure 4:**
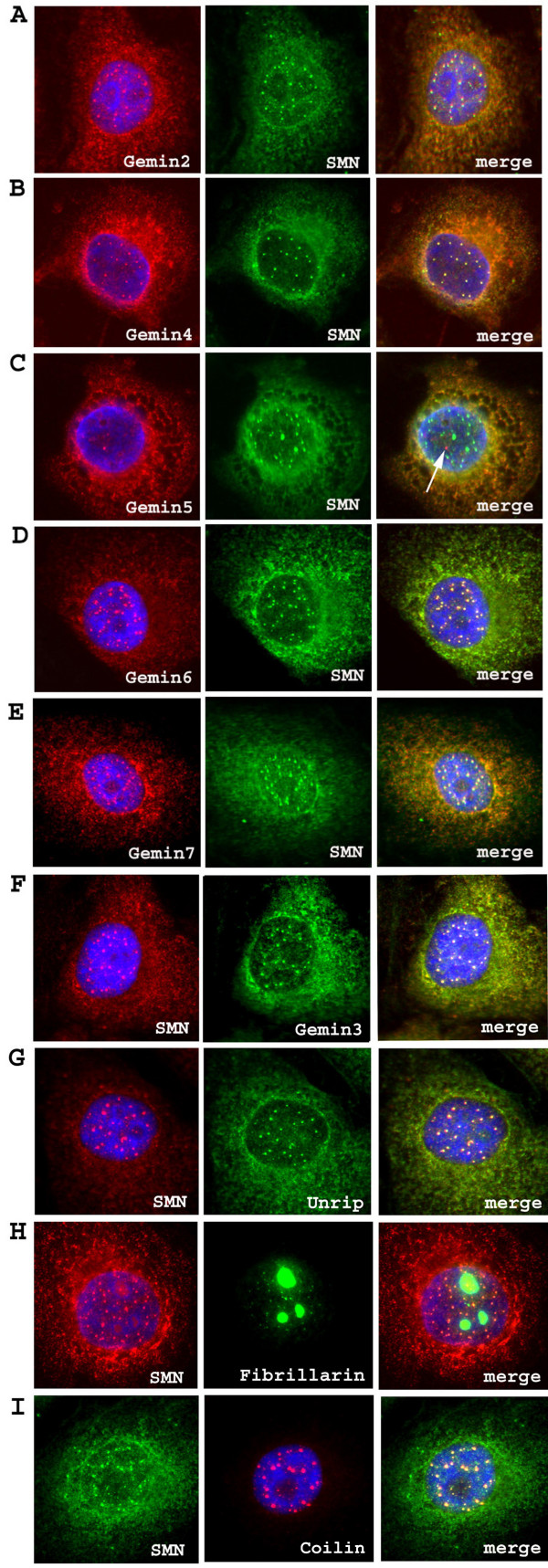
**Overexpression of SMN in HeLa cells stimulates formation of gems/CBs containing other gemins, except gemin 5**. Gemin2 (A), gemin 4 (B), gemin6 (D) and 7 (E) co-localise with SMN in HeLa cells stably transfected with human SMN1 in pcDNA3 [49]. Gemin5 (C) does not co-localise with SMN. The same mAbs as in Fig. 2 were used, plus MANSIP1A for gemin2. Double label of SMN mAb MANSMA1 with gemin3 rabbit antiserum (F), unrip rabbit antiserum (G) and fibrillarin human auto-antiserum (H), or SMN antiserum with p80 mAb (I) were performed on the HeLa cell line stably transfected with SMN1. Alexa fluor 546 goat anti-mouse IgG (red) was used to detect mouse mAbs and Alexa fluor 488 goat anti-rabbit IgG (green) was used to detect rabbit antibodies. FITC-conjugated goat anti-human IgG (Chemicon) was used to detect fibrillarin. DAPI (blue) was used to counterstain the nuclei.

SMN-containing gems/CBs are increased in number and brightness in a HeLa cell line overexpressing SMN protein, so we set out to determine whether antibody staining of these structures for gemins was also increased. Gemins 2, 3, 4, 6 and 7 did colocalize with SMN in these up-regulated gems/CBs, but gemin5 was still not detected in these structures (Fig. 4ab). Fig. 4b shows that unrip also colocalises with the up-regulated gems/CBs and that traces of fibrillarin, a predominantly nucleolar protein, can also be detected in the up-regulated gems/CBs (at such low levels, however, the possibility of a slight cross-reaction of this auto-antiserum cannot be entirely ruled out). Fig. 4b also confirms earlier evidence [31] that coilin, the marker for Cajal bodies, is up-regulated in gems/CBs when SMN levels are increased by transfection.

**Figure 5:**
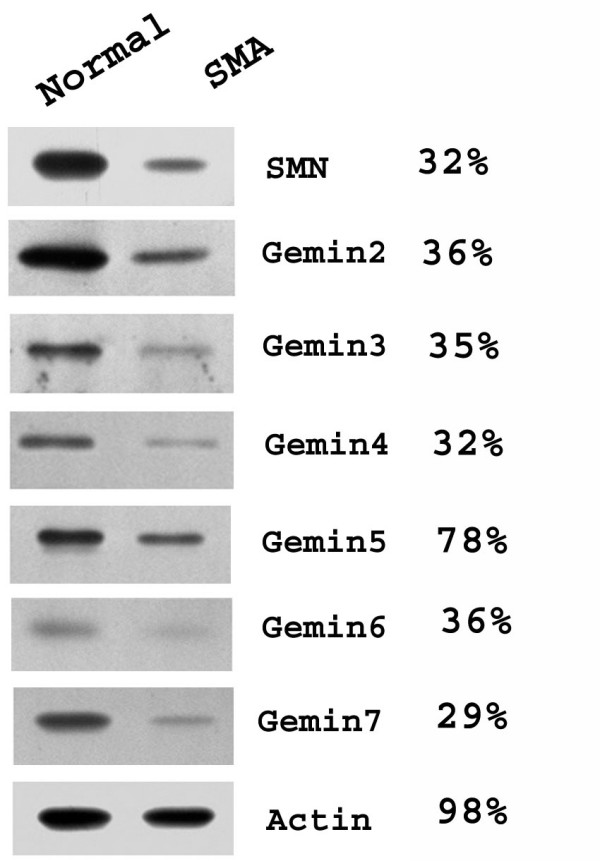
**All gemins, except gemin5, are proportionately reduced in SMN-deficient human skin fibroblasts**. Total SDS extracts of the two skin fibroblast cell lines (Coriell cell lines, GM08333, control and GM03813, SMA) were subjected to SDS-PAGE on adjacent lanes of a 7% or 14% acrylamide gel (cf. Fig. 1), followed by western blotting with the same antibodies used in Fig. 2 plus MANSIP1A for gemin2. β-actin was used as a control for equal loading of the gel lanes. Microdensitometry was used to express protein levels in SMA fibroblasts as a percentage of those in control fibroblasts.

**Figure 6:**
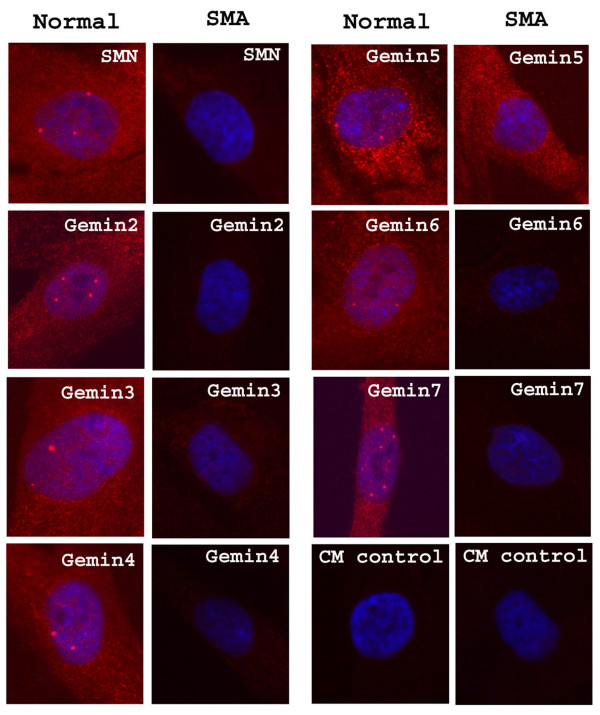
**Nuclear gems/CBs and cytoplasmic levels of most gemins are greatly reduced in SMN-deficient human skin fibroblasts, but cytoplasmic gemin5 levels remain high**. Alexa fluor 546 goat anti-(mouse IgG) (red) was used to detect SMN and gemin 2, 4, 5, 6 and 7 primary mAbs (the same mAbs as in Fig. 2). TRITC donkey anti-(rabbit Ig) was used to detect anti-gemin3 rabbit antibodies. DAPI (blue) was used to counterstain the nuclei.

SMN on western blots was decreased to 30–40% of control levels in cultured skin fibroblasts derived from an SMA type I patient (Fig. 5). Most gemins were also decreased by a similar proportion, but gemin5 showed a smaller reduction (Fig. 5). These reductions in SMA fibroblasts were also evident by immunofluorescence microscopy (Fig. 6). SMN and most gemins showed drastically decreased levels in both nuclear gems and cytoplasm in SMA fibroblasts. Gemin5, however, remained stable in the cytoplasm, consistent with the western blot data in Fig. 5 and with the existence of a separate pool of gemin5, independent of SMN.

### Unlike other gemins, gemin5 is present mainly in the cytoplasm

**Figure 7:**
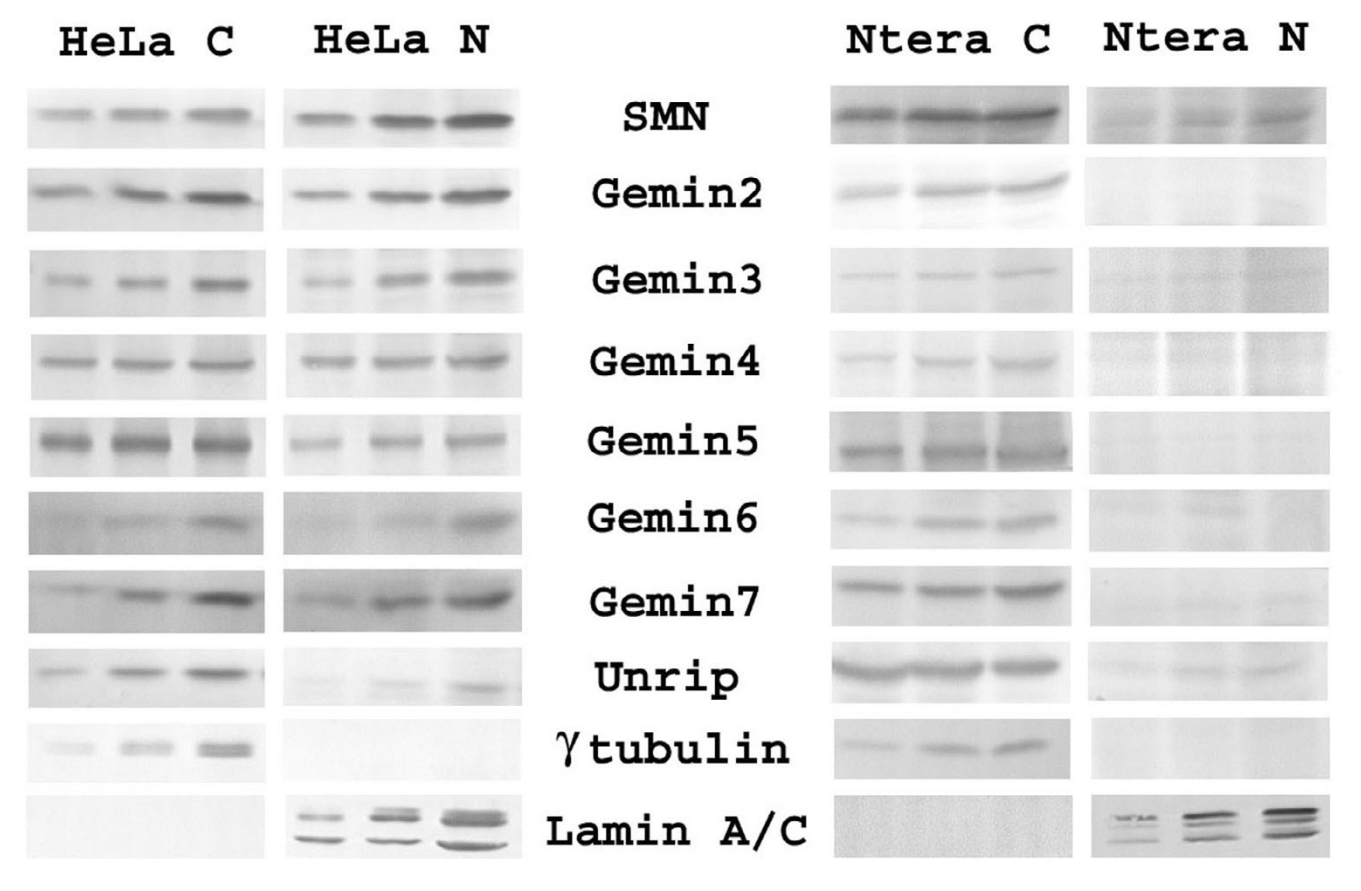
**Distribution of gemins and other proteins between cytoplasmic and nuclear fractions of HeLa and Ntera2 cells**. HeLa C and Ntera C are cytoplasmic fractions, HeLa N and Ntera N are nuclear fractions. After extraction with cytoplasmic buffer (see Methods), the whole pellet was boiled in SDS sample buffer to obtain the "nuclear" extract. Samples were loaded as serial dilutions (from left to right, 1/4, 1/2 and 1) for more accurate microdensitometry quantitation. γ tubulin and lamin A/C were used as controls for cross-contamination between nuclear and cytoplasmic fractions.

We next examined the distribution of SMN and gemins between nucleus and cytoplasm using a simple cell fractionation followed by western blotting (Fig. 7). To minimise quantitation problems with X-ray film exposure, we used 100%, 50% and 25% dilutions of each extract and compared the closest match by microdensitometry. Comparisons can only be reliably made between fractions of the same protein, and not between different proteins, because of possible differences in antibody avidity. In HeLa cells, SMN and most gemins were equally distributed between nucleus and cytoplasm, but gemin5 was predominantly cytoplasmic. This could mean that gemin5 has additional SMN-independent functions in the cytoplasm, but it is also possible that gemin5 is turned over in, or exported from, the nucleus more quickly than other gemins. HeLa cells are widely used for SMN studies but it would be unwise to assume that they are representative of human cells in general. In the neurogenic cell line, Ntera-2, we found that SMN and gemins were much more cytoplasmic than in HeLa cells. In both skin fibroblasts and Ntera-2 cells, gems/CBs were rarely, if ever, stained by anti-gemin5 mAbs (data not shown).

### On sucrose density gradient analysis, gemin5 is absent from SMN complexes in nuclear extracts, but not in cytoplasmic extracts

**Figure 8:**
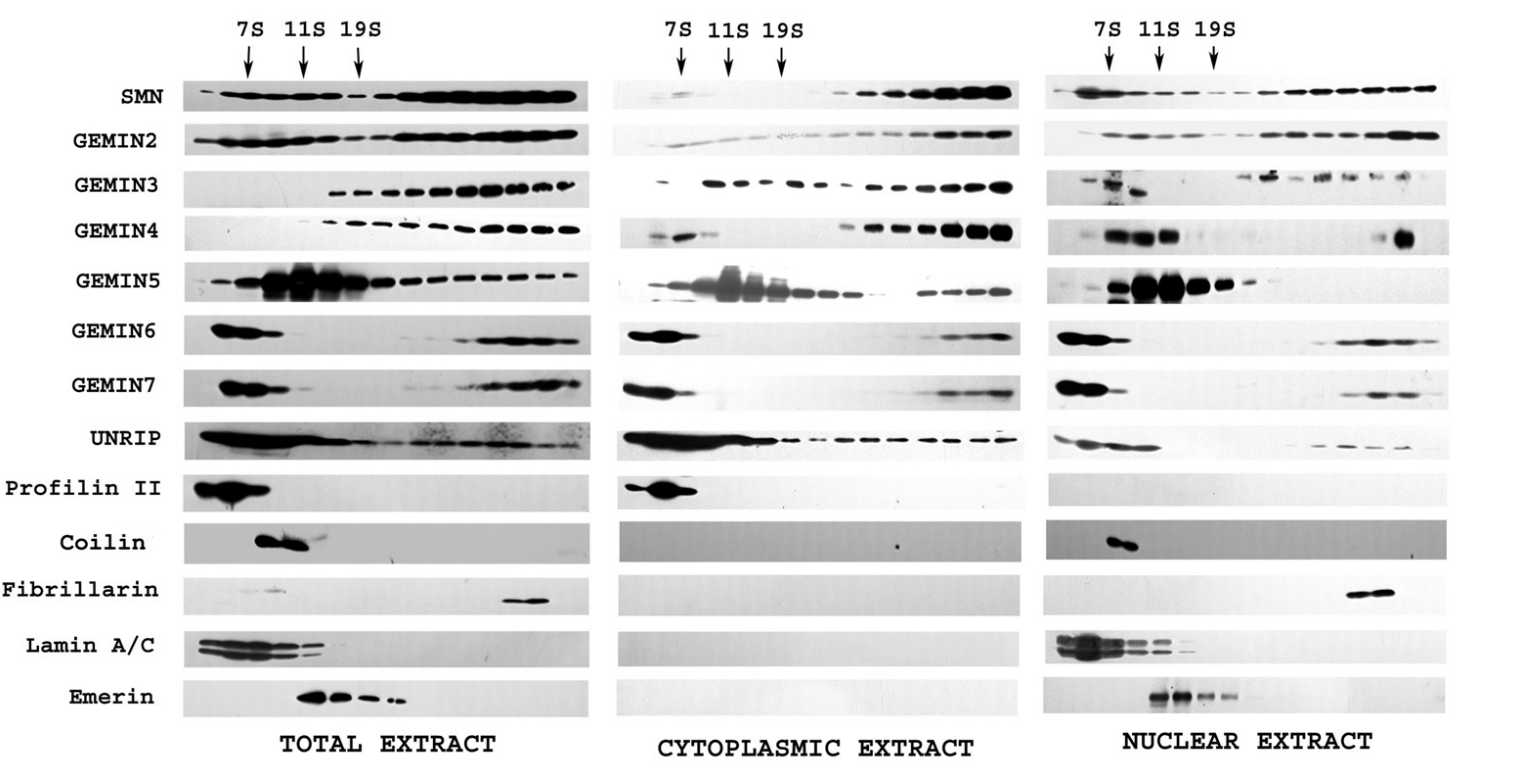
**Distribution of SMN and gemins between large, fast-sedimenting SMN complexes and slower sedimenting fractions**. HeLa total, cytoplasmic and nuclear extracts were prepared and analyzed by centrifugation on 15–30% sucrose density gradients. Thirty 1 ml fractions were collected from each gradient and the protein contents were concentrated into 0.1 ml using Strataclean resin. Every other fraction (1, 3, 5, etc) was subjected to SDS-PAGE for western blotting using appropriate antibodies. The size markers were immunoglobulin (7S), catalase (11S) and thyroglobulin (19S).

The extent to which gemin5 and other gemins are present in complexes with SMN was studied by sucrose density gradient centrifugation. We analysed a total extract of HeLa cells prepared using a RIPA buffer and an extract separated into nuclear and cytoplasmic fractions using the established method of Meister et al [20]. These experiments were repeated three times starting with fresh HeLa cells and a representative result is shown in Fig. 8. It seems likely that some detachment of proteins from the SMN complexes occurs during cell lysis or fractionation with the harsher RIPA or high-salt nuclear buffers. Thus, no SMN or gemin2 is seen at the top of the gradient in low-salt cytoplasmic extracts (Fig. 8b) but they are present when RIPA buffer (Fig. 8a) or higher salt (Fig. 8c) are used. The mild extraction used for the cytoplasmic fraction is more likely to yield intact SMN complexes (Fig. 8b) but, although most of the SMN and gemins 2, 3 and 4 sedimented in large complexes, the majority of both gemin5 and unrip remained in the upper part of the gradient (<19S), together with a significant proportion of gemins6/7. This would be consistent with these latter proteins having some additional functions outside SMN complexes.

Successful subcellular fractionation is shown by the presence of nuclear markers, such as lamin A/C, emerin, coilin p80 and fibrillarin, only in the nuclear fraction while profilin II was present only in the cytoplasmic fraction. As expected, all gemins and unrip are partly present in large complexes migrating near the bottom of the sucrose gradient, whereas other nuclear protein markers, emerin and lamin A/C, remained in the upper half of the gradient. Fibrillarin is clearly sedimenting in high molecular weight complexes but since most fibrillarin is in the nucleolus and very little colocalizes with SMN, this material seems unlikely to represent complexes with SMN. SMN, however, does colocalize with fibrillarin in the nucleolus in fetal tissues [34] and the possibility that SMN is complexed with fibrillarin in a "masked" form [35] in HeLa nuclei has never been ruled out. The proteins in the upper half of the gradients would be consistent in size with monomers or small homo- or hetero-oligomers. Thus, gemins 6 and 7 (15–19 kD) are in the first two fractions, while SMN and gemin2 (32–38 kD) co-sediment close to the 7S marker. Gemin5 (167 kD) monomers would be expected around 7S and its faster sedimentation at around 11S might be consistent with a proposed trimeric structure [29].

**Figure 9:**
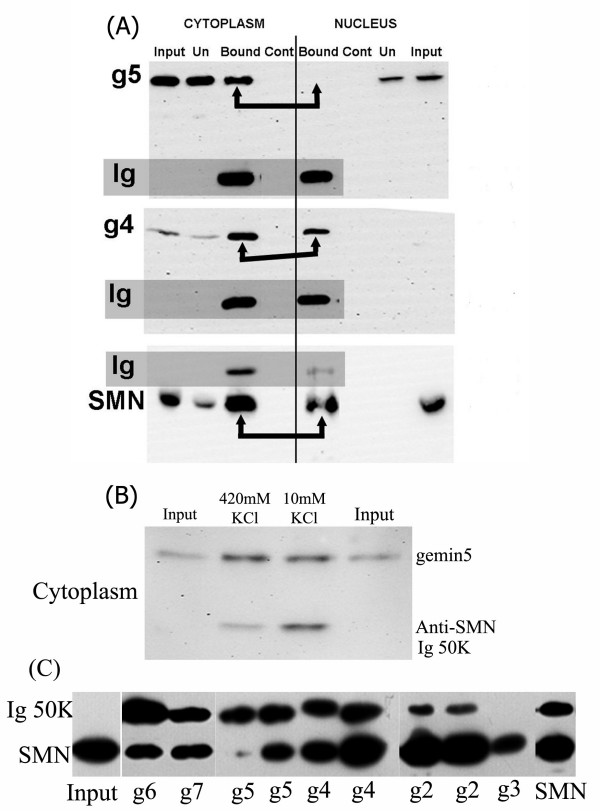
(A) Co-immunoprecipitation with MANSMA1 anti-SMN mAb shows that SMN and gemin5 exist as complexes in cytoplasmic extracts, but not in nuclear extracts (arrows show lanes to be compared). SMN and gemin4 controls are pulled down from both extracts equally. (B) Addition of KCl to the cytoplasmic extract to the same concentration as nuclear extraction buffer did not disrupt the SMN-gemin5 interaction. (C) All gemins in SMN complexes were accessible to appropriate antibodies. All anti-gemin antibodies pulled down SMN from total RIPA extracts of HeLa cells, except GEM5G which only recognizes denatured gemin5 on western blots (this also acts as a negative control). Antibodies were (left to right; see Table 1): No mAb (input control), GEM6B, GEM7B, GEM5G, GEM5P, GEM4C, GEM4D, MANSIP1B, MANSIP1A, rabbit anti-gemin3 and MANSMA1. In each case, 0.05 ml of Dynabead magnetic beads (Dynal, Oslo, Cat. No. 100.41), with anti-mouse Ig (or anti-rabbit Ig) attached covalently, were incubated with 0.1 ml of undiluted mAb culture supernatant (or 1/100 dilution antiserum) for 1 h at 4°C, washed 3× with PBS containing 0.1% BSA, and then incubated for 16 h at 4°C with 0.08 ml of HeLa extract (also sampled as "input"). After removing the "unbound" extract, the beads were washed 5× with PBS and boiled in 0.02 ml of SDS sample buffer. Gels (10% or 12.5% polyacrylamide) were loaded with 0.01 ml of SDS extract for SDS-PAGE and western blotting with anti-SMN mAb MANSMA12, as described in Methods. All lanes loaded with SDS extracts of beads contain a 50 KDa band of mouse Ig heavy chain which reacts with the HRP anti-mouse Ig used to develop the blot (band is absent from input and when rabbit antisera are used on beads).

Gemin5 did not co-sediment at all with the large SMN complexes in the nuclear extract (Fig. 8c), consistent with its absence from gems/CBs (Fig. 2). However, the possibility that gemin5 detached from the complexes during fractionation cannot be ruled out. In a recent study, gemin5 was more easily detached by high salt than other gemins [2], though this occurred at higher salt concentrations (>500 mM) than those used for nuclear extraction (440 mM total). Adding extra KCl to cytoplasmic extracts to 440 mM did not dissociate gemin5 from SMN (see Fig. 9b below), but the possibility that nuclear complexes are less stable cannot be ruled out. Therefore, we have restricted our major conclusions to the clear invivo reduction of gemin5 in nuclear gems/CBs by immunolocalization studies (Fig. 2), which cannot be explained by fractionation artefacts, and to the complexes present under mild extraction conditions (Fig. 8b).

### Gemins co-sedimenting on sucrose gradients form true complexes in which all gemins are accessible to antibody

Immunoprecipitation experiments were performed to demonstrate true complex formation, as opposed to co-sedimentation on the sucrose gradients. MANSMA1 anti-SMN mAb pulled down gemin5 from cytoplasmic complexes only, in agreement with sucrose gradient data (Fig. 8), whereas it pulled down SMN itself and the gemin4 control from both nuclear and cytoplasmic complexes (Fig. 9a). To determine whether the absence of gemin5 from SMN complexes in nuclear extracts was due to disruption by high salt, cytoplasmic extracts were studied after adding KCl to the same concn as the nuclear extracts and gemin5 was still pulled down by the anti-SMN mAb (Fig. 9b). To show that all gemins are accessible to antibody in SMN complexes, the immunoprecipitation experiment was reversed and various anti-gemin mAbs were used to pull-down endogenous SMN protein from a total HeLa extract in RIPA buffer. This extract contains both nuclear and cytoplasmic complexes, and all gemin mAbs, including gemin5, pulled down SMN (Fig. 9c). The predictable exception was a mAb that only recognizes denatured gemin5 on blots and would not be expected to bind native complexes. This experiment confirms that gemin5 in SMN complexes is as accessible to antibody as the other gemins and shows that masking of gemin5 epitopes cannot explain the absence of gem/CB staining by gemin5 mAbs in immunofluorescence microscopy (Fig. 2).

### Discussion and conclusion

The idea that SMN complexes may vary in composition is not a novel one. Dreyfuss and co-workers have explained their own data in terms of a range of structures from SMN-gemin2 heterodimers upwards [36]. It is also implicit in the studies of Meister et al [20,21]. The possibility that compositional changes may be relevant to understanding the function of SMN complexes has not, however, been fully explored, partly because the roles of the SMN complex and its individual protein components are still not fully understood and partly because of the uncertainty about the stability of complexes after cell disruption for biochemical studies.

In the present study, sucrose gradient analysis has shown that SMN complexes in nuclear extracts have little or no gemin5. This was also noted in an earlier study [27]. Pull-down experiments with anti-SMN mAb confirmed that SMN is associated with gemin5 in cytoplasmic extracts but not in nuclear extracts. A major problem with subcellular fractionation, however, is the difficulty in ruling out possible dissociation during biochemical fractionation, with some components of SMN complexes being more easily dissociated than others. In our hands, raising the salt concn in cytoplasmic extracts to 440 mM (same as nuclear extracts) did not noticeably dissociate gemin5 from SMN and this agrees with a recent study showing that a higher concentration (>500 mM) is required to do this [2]. Even so, we cannot rule out the possibility that some dissociation of SMN complexes has occurred in nuclear extracts.

Therefore, the observation in intact cells that gemin5 does not colocalize with SMN and all other gemins in nuclear gems/CBs is important to show that its absence from complexes is not just an artefact of biochemical fractionation. It is significant that gemin5 was found in gems/CBs in a very small proportion of HeLa cells (<1%) and that, when present, it was present in all gems/CBs of that nucleus. This is a key observation since it shows that gemin5 is easily detected by immunofluorescence microscopy, when it is present. It also suggests that these minority cells may differ metabolically or phenotypically from the majority. The possibility that binding of mAbs to gemin5 was masked by other components of the SMN complex is ruled out by the fact that the same gemin5 mAbs are as capable of immunoprecipitating SMN as mAbs against other gemins (Fig. 9c). Overexpression of SMN in a stably-transfected HeLa cell line increases the number and brightness of nuclear gems/CBs when stained with SMN antibodies or with most other gemin antibodies, but it did not bring about increased immunostaining of gemin5 in gems/CBs, which we might expect if the gemin5 mAbs were simply rather weak in immunostaining. We hypothesize that the most important role of gemin5 is in the cytoplasmic part of the SMN cycle and that its role becomes less important once the assembled snRNPs have been transported to the nucleus by the SMN complex. Gubitz et al [29] have suggested that the WD propeller structure of the gemin5 N-terminal domain may act as an assembly platform for other proteins and recent reports suggest that gemin5 is involved in binding the snRNA component for snRNP assembly [37].

It is also of interest that, even in the cytoplasm, a significant proportion of the gemin5 is unassociated with SMN complexes. This is evident both from the sucrose gradients in Fig. 8b and from the fact that both cytoplasmic gemin5 staining and gemin5 levels on western blots remain strong in type I SMA fibroblasts, when SMN and other gemins are clearly reduced by 60–70% relative to control fibroblasts. Similarly, RNAi knockdown of SMN had comparatively little effect on gemin5 levels (cf. Fig. 2A of Feng et al, [38]). Feng et al [38] also showed that RNAi knockdown of gemin5 did not affect gems/CBs or the levels of other SMN complex components on western blots. Similarly, Shpargel and Matera [39] found that RNAi knockdowns of SMN or gemins 2, 3 or 4 all disrupted Sm core protein assembly, whereas knockdown of gemin5 had no effect. These observations are consistent with our data, though the presence of a larger cytoplasmic pool of "free" gemin5 could mean that a much more efficient knockdown of gemin5 would be required to obtain the same result as other gemin knockdowns.

An excess of "free" gemin5 over that required for SMN complexes might imply a more general role for gemin5. An interaction with eukaryotic initiation factor 4E has recently been described and a role for gemin5 in assembly or processing of messenger RNP complexes was suggested [40]. In their study, mass spectrometry did not reveal SMN or any other gemins in these gemin5 complexes. The possibility that gemin5 may have a more general role in RNP assembly was raised by Gubitz et al [29]. Unrip is another WD-repeat protein, like gemin5, and it also has a clearly-demonstrated role in mRNA translation initiation [22]. A further role for unrip in assembling mRNA export and transport intermediates has recently been proposed [41]. Carissimi et al [23] and Grimmler et al [42] also found that unrip, like gemin5 in the present study, was predominantly cytoplasmic and not localized in nuclear gems/CBs. Our results confirm the mainly cytoplasmic distribution of unrip (Fig. 8), but a small amount of unrip in the nuclear fraction was present in large complexes (Fig. 8) and was detectable in nuclear gems/CBs of HeLa cells overexpressing SMN (Fig. 4G). These differences between laboratories could be due to antibody avidity or SMN expression levels and a consensus may be that both of these two WD-repeat proteins, gemin5 and unrip, are present at relatively low, often undetectable, levels in gems/CBs. It may be that both proteins are involved in assembling complexes of SMN with RNA and other proteins but are no longer essential when the complexes arrive in nuclear gems/CBs.

Although SMN and profilin II interact in vitro and are both detectable in nuclear gems/CBs in neuronal cells [17], we found no evidence for the presence of profilin II in the SMN-containing complexes (Fig. 8), possibly because the interaction is weaker and profilin I may be the dominant isoform in HeLa cells. Coilin was not found in the large SMN complexes, although a methylated form can interact with SMN [43] and it does colocalize with nuclear SMN in gems/Cajal bodies in most HeLa cell lines. However, SMN and coilin p80 can also form quite separate gems and Cajal bodies in fetal tissues [34] and coilin p80 is exclusively nuclear, so the result is not altogether surprising. SMN complexes involve so many proteins, even the "core" complex let alone ancillary interactors, that much work remains to be done in elucidating what exactly is binding to what under different cellular conditions [1,2]. There is evidence, for example, that the activity of SMN in a snRNP assembly assay can vary developmentally without changing SMN levels [44], though it is not known whether this requires any changes in SMN complex components. The present study suggests that gemin5 works only "part-time" as a component of SMN complexes, in the sense that they can exist without gemin5 and that gemin5 may have functions that are independent of SMN complexes, though possibly involving related assembly functions. In some ways, the behaviour of gemin5 resembles that of another WD-repeat protein, unrip, as reported either in this study or elsewhere.

## Methods

### Cloning and expression of gemin cDNAs

Total RNA was isolated from HeLa cells using an RNAeasy Midi Kit (Qiagen) and converted to cDNA using Sensiscript reverse transcriptase (Qiagen) and random hexamer primers. Specific gemin cDNA sequences were obtained by PCR using the primers shown in Table 3. The primers contained restriction sites for cloning into pET21b or pET32b; EcoRI and XhoI for gemin4, BamHI and HindIII for gemin5 and BamHI and XhoI for gemins 6 and 7. The gemin3 PCR product was cloned into the pT7Blue plasmid, cut from it using the plasmid BamHI and XhoI sites and subcloned into the same sites of pET21b. The cDNAs encoded aa 18–362 of gemin3, the first 650aa of gemin4, aa55–407 for gemin5 N-terminal region, aa 1217–1508 for gemin5 C-terminal region and full-length for gemins 6 and 7. All constructs were sequenced to check for PCR errors. For immunogen production, gemins 3, 4 and N-terminal gemin5 were expressed from pET21b and the rest from pET32b.

**Table 3:** Oligonucleotide primers for PCR

Primer	Primer sequences (5'-3')
Gemin6F	ga**g gat cc**c atg agt gaa tgg atg aag
Gemin6R	gc**c tcg ag**t cat tgg gaa gct gta aga
Gemin7F	ga**g gat cc**a atg caa act cca gtg aac
Gemin7R	gc**c tcg ag**t tat ggc ttg aag gta ta
Gemin3F	agt gac cta ctg ttg ccg g
Gemin3R	cat ctt cct ggg ctt cag tgc
Gemin 4F	atc gc**g aat tc**c atg gac cta gga ccc ttg
Gemin 4R	ata att **ctc gag **cag cac ctc gtc tgg ctc
Gemin 5FN	gca ct**g gat cc**t cga gtc ata gga gag ttg
Gemin 5RN	gct cgg **aag ctt **cca tac acg gat cat gcc
Gemin 5FC	aat ta**g gat cc**g atg gcc tcc tgg gac gag
Gemin 5RC	gcg cgg **aag ctt **cat aca gaa ggt ctg gca

Transformed bacteria [E. coli BL21(DE3)] were induced with 1 mM IPTG for 3 h at 37°C. Expressed fusion protein was purified from inclusion bodies by sequential extraction with increasing concentrations (2 M, 4 M, 6 M, 8 M) of urea in phosphate-buffered saline (PBS). The extracts in 6 M urea were then purified by affinity chromatography with His. Bind resin (Novagen).

### Production of antibodies

Monoclonal antibodies were produced by immunization of BALB/c mice and fusion of spleen cells with Sp2/0 myeloma cells as described elsewhere [45]. Both the mouse sera and the hybridoma culture supernatants were screened by elisa, Western blot (HeLa total protein extract) and immunofluorescence microscopy (nuclear gems/CBs in HeLa cells). Hybridoma cell lines were cloned to homogeneity by limiting dilution. Ig subclass was determined using an isotyping kit (Zymed, San Francisco). Polyclonal antibodies were produced commercially by immunization of a New Zealand White rabbit with recombinant protein (Harlan SeraLab, Loughborough, UK).

Other antibodies used in this study were rabbit antiserum against SMN and mAbs MANSMA1 and MANSIP1A against SMN and gemin2 [31], mAb 5P10 against coilin p80 (gift of Angus Lamond, University of Dundee, UK [46]), human autoantiserum against fibrillarin (gift of K. Michael Pollard, Scripps, San Diego [33]), mAb MANEM5 against emerin [47], mAb MANLAC1 against lamin A/C [48], mAb PF2A3 against profilin II and rabbit antiserum against unrip [17], mAb 8226 against beta-actin and rabbit antiserum 11317 against gamma-tubulin (Abcam, Cambridge, UK).

### SDS-PAGE and western blotting

SDS-PAGE and western blotting were carried out as described elsewhere [17]. Protein bands were visualized by development with peroxidase-conjugated rabbit anti-(mouse Ig) (DAKOpatts) and a chemiluminescent system (SuperSignal; Pierce). Quantitation was performed by microdensitometry of captured blot images using Laserpix software (BioRad Laboratories). To minimize problems with non-linear response of X-ray film, several dilutions of cell extracts were blotted and those that gave the same, or similar, intensities in a linear response region were used for comparative quantitation.

### Immunohistochemistry

HeLa, Ntera-2 (NT-2) and skin fibroblast (GM08333, control and GM03813, SMA from Coriell Cell Bank, Camden, NJ) cell lines and the stably-transfected SMN-overexpressor fibroblast cell line were grown on coverslips in DMEM with 10% horse serum and fixed with 1% formalin or 50:50 acetone-methanol. As shown previously [49], the levels of recombinant SMN over-expression in the stably-transfected cell line were similar to endogenous SMN levels, thus approximately doubling total SMN levels. Formalin-fixed cells were permeabilized with 1% Triton X-100 and blocked with 1% glycine before use. For double labeling experiments, coverslips were incubated with the mouse mAb for 1 h, followed by the rabbit polyclonal antibody for 1 h. Alexa488- or Alexa546-conjugated goat anti-(mouse Ig) and goat anti-(rabbit Ig) (Molecular Probes, Eugene, OR) were then applied. Nuclei were revealed with a DAPI counter stain and slides were viewed using the L4 filter set and a 63× oil immersion objective on a Leica DMRB photomicroscope. Images were captured using an integrating camera and frame-grabber under standard and comparable conditions.

### Subcellular fractionation studies

300–600 ml of HeLa cells were grown in suspension culture for 4 days and washed in ice-cold PBS. The pellet was resuspended in 4 volumes of RIPA buffer (1% NP40, 0.25% deoxycholate, 1 mM EDTA, 1 mM PMSF, 150 mM NaCl, 50 mM Tris-HCl pH 7.4), left on ice for 30 minutes, homogenizing every 10 minutes. The homogenate was centrifuged at 13,000 g for 15 minutes at 4°C and the supernatant retained. Cytoplasmic and nuclear fractions were prepared as described by Meister et al [20]. Briefly, HeLa cells were homogenized in 10 mM KCl, 10 mM HEPES-KOH pH 7.9, 1.5 mM MgCl_2_, 0.5 mM PMSF and 0.5 mM dithiothreitol and centrifuged for 10 minutes at 1000 g at 4°C. The supernatant was clarified further at 13,000 g for 10 minutes at 4°C to produce the cytoplasmic fraction. The nuclear pellet was homogenized in 1 ml of 420 mM KCl, 20 mM HEPES-KOH pH 7.9, 1.5 mM MgCl_2_, 0.5 mM PMSF, 0.2 mM EDTA and 5% glycerol, left for 30 minutes on ice and then centrifuged 13,000 g for 30 minutes at 4°C. The supernatant was designated as the nuclear fraction.

RIPA extracts or cytoplasmic and nuclear fractions were analysed on 35 ml 15–30% sucrose density gradients in 150 mM NaCl, 50 mM Tris HCl pH 7.4 and 5 mM MgCl_2 _by centrifugation in a Beckmann SW28 rotor at 25,000 g for 21 hours at 4°C. Thirty fractions were collected and every other fraction was concentrated 10-fold using Strataclean resin (Stratagene, Amsterdam, Netherlands) and analyzed by SDS-PAGE and western blotting.

## Authors' contributions

LTH carried out most of the laboratory studies and helped to draft the manuscript. HRF designed and performed the HeLa PV and immunoprecipitation studies. LTL and TTL produced DNA constructs and cell lines for the study. AHMB participated in the design of the study and helped to draft the manuscript. GEM conceived of the study, participated in its design and coordination, contributed to hybridoma production and helped to draft the manuscript. All authors read and approved the final manuscript.
